# Shooting shadows: India’s struggle to reduce the burden of anaemia

**DOI:** 10.1017/S0007114522000927

**Published:** 2023-02-14

**Authors:** Rajesh Kumar Rai, Sandhya S. Kumar, Sourav Sen Gupta, Devraj J. Parasannanavar, Thekkumkara Surendran Nair Anish, Anamitra Barik, Rajeev Kumar Varshney, Hemalatha Rajkumar

**Affiliations:** 1 Department of Economics, University of Goettingen, Goettingen, 37073, Germany; 2 Centre for Modern Indian Studies, University of Goettingen, Goettingen, Germany; 3 Society for Health and Demographic Surveillance, Suri, West Bengal, India; 4 Department of Global Health and Population, Harvard T H Chan School of Public Health, Boston, MA, USA; 5 World Vegetable Center, South and Central Asia, Hyderabad, Telangana, India; 6 Indian Institute of Liver and Digestive Sciences, Liver Foundation, Kolkata, West Bengal, India; 7 International Crops Research Institute for the Semi-Arid Tropics, Hyderabad, Telangana, India; 8 Division of Clinical Epidemiology, Indian Council of Medical Research, National Institute of Nutrition, Hyderabad, Telangana, India; 9 Department of Community Medicine, Government Medical College Thiruvananthapuram, Thiruvananthapuram, Kerala, India; 10 Suri District Hospital, Suri, West Bengal, India; 11 State Agricultural Biotechnology Centre, Centre for Crop and Food Innovation, Food Futures Institute, Murdoch University, Murdoch, WA, Australia; 12 Indian Council of Medical Research, National Institute of Nutrition, Hyderabad, Telangana, India

**Keywords:** Anaemia, Iron deficiency anaemia, Micronutrients, Iron supplements, Food policy

## Abstract

Despite several efforts by the Government of India, the national burden of anaemia remains high and its growing prevalence (between 2015–2016 and 2019–2021) is concerning to India’s public health system. This article reviews existing food-based and clinical strategies to mitigate the anaemia burden and why they are premature and insufficient. In a context where multiple anaemia control programmes are in play, this article proposes a threefold strategy for consideration. First, except the Comprehensive National Nutrition Survey, 2016–2018, which measured Hb concentration among children and adolescents aged 1–19 years using venous blood samples, all national surveys use capillary blood samples to determine Hb levels, which could be erroneous. The Indian government should prioritise conducting a nationwide survey for estimating the burden of anaemia and its clinical determinants for all age groups using venous blood samples. Second, without deciding the appropriate dose of Fe needed for an individual, food fortification programmes that are often compounded with layering of other micronutrients could be harmful and further research on this issue is needed. Same is true for the pharmacological intervention of Fe tablet or syrup supplementation programmes, which is given to individuals without assessing its need. In addition, there is a dire need for robust research to understand both the long-term benefit and side effects of Fe supplementation programmes. Third and final, the WHO is in process of reviewing the Hb threshold for defining anaemia, therefore the introduction of new anaemia control programmes should be restrained.

India has the largest number of anaemic populations in the world^(1–5)^, and an increasing trend (between 2015–2016 and 2019–2021) of its prevalence has drawn concern and criticism from global public health researchers, donors and policy makers. Clinically, anaemia is defined as the reduction in Hb concentration, red blood-cell count or packed-cell volume below an established threshold^(5)^. As per current guidelines, a Hb concentration of <11 g/decilitre (g/dl) among children (aged 6–59 months) and pregnant women (aged 15–49 years) is considered anaemic, whereas non-pregnant women with a Hb of <12 g/dl and men (aged 15–49 years) with a Hb of <13 g/dl are labelled anaemic^(6)^. Physiologically, the anaemic condition impairs the body’s ability to transport oxygen (from lungs to tissue) and carbon dioxide (from tissue to lungs), which could be fatal^(6)^. The adverse effects of anaemia are well documented, and they vary with different stages of life^(7)^. While anaemia can cause morbidity and mortality in all age groups, irrespective of sex, it affects women disproportionately, especially those in the reproductive age group of 15–49 years^(8–12)^. Anaemia can also impair cognitive and behavioural development among children and adolescents^(4,13–15)^ and decreases work productivity among adult men and women^(16)^, causing a huge economic loss to the country’s productivity^(16)^. Anaemia among older adults is less well documented, but it is known that the prevalence of anaemia among adults aged over 50 years rises with advancing age, particularly among men^(17)^.The aetiology of anaemia is complex and factors that contribute to it go beyond Fe deficiency^(18)^ to include inadequate intake of other nutrients such as vitamins A^(19)^, B_6_, B_12_^(20,21)^, C, D and E^(22,23)^, folate^(22)^, Zn^(24–26)^ and Cu^(27)^; exposure to disease and infections (soil-transmitted helminth infections, schistosomiasis, malaria, human immunodeficiency virus, tuberculosis and low-grade inflammation)^(17)^ and genetic haemoglobinopathies^(7)^ among other factors^(17,28,29)^. While clinical causes of anaemia are documented widely, the social, behavioural and environmental determinants of anaemia are equally important. The 2016 Global Burden of Disease group modelled Fe-deficiency anaemia (IDA) to be the top cause of years lived with disability in India^(3)^. Unfortunately, India lacks recent national estimates on clinical causes of anaemia for all age groups of adult men, women and children. While regional and local surveys and estimates are helpful in gaining a perspective of nutritional and non-nutritional causes of anaemia, these studies are insufficient for devising national programmes and policies.

The burden of anaemia varies widely across states and union territories in India (Table 1). According to the nationally representative 2015–2016 National Family Health Survey (NFHS) of India^(30)^, over 50 % of its women and children suffered from anaemia, and a meagre reduction of anaemia was registered between 2005–2006 and 2015–2016 (Table 1, and Fig. 1) among children^(30)^, adult men^(2)^ and women^(31)^. Although the anaemia prevalence decreased between 2005–2006 and 2015–2016, the recent estimate from 2019–2021 NFHS^(32)^ showed an increase of anaemia (Table 1, and Fig. 1), indicating a pervasive public health system failure requiring detailed scrutiny of all anaemia control programmes and policies. NFHS is a nationally representative cross-sectional survey conducted under the stewardship of Ministry of Health and Family Welfare, Government of India. From the predefined sampling frame of census of India, NFHS selects primary sampling units in rural and urban areas and adopts a stratified two-stage sampling design. Primary sampling units are identified as villages in rural areas and Census Enumeration Blocks in urban areas. Within each rural stratum, villages are selected from the sampling frame with probability proportional to size^(30,31)^. By virtue of sampling design, estimates across NFHS rounds are comparable^(33)^. The primary data on Hb concentration obtained from the survey participants were adjusted for the smoking or/and altitude of living^(34)^. According to the 2016–2018 Comprehensive National Nutrition Survey (CNNS) report, 28 % of India’s adolescents aged 10–19 years had some degree of anaemia, and 12 % of anaemic respondents were Fe deficient^(35)^. However, except estimates from CNNS, estimates of anaemia from NFHS should be interpreted with caution as it used the HemoCue device to measure anaemia, which remains debatable for its accuracy. HemoCue is a portable device and uses the capillary blood sample to detect anaemia which tends to overestimate Hg levels compared with measuring with a venous blood sample^(36)^. All rounds of the NFHS used a comparable model of the HemoCue device to measure Hb level. A study in Cambodia^(37)^ found an overall bias in Hg concentration of 2·6 g/l, using HemoCue 201+ which resulted in a difference in anaemia prevalence of 11·5 %. In addition, a recent study conducted among children and adolescents aged 1–19 years in India^(38)^ found an average bias for the venous HemoCue and capillary HemoCue were 3·0 ± 4·0 and −3·0 ± 11·0, respectively. Thus, it is evident that India lacks a true estimate on the prevalence of anaemia for all age groups^(39)^, and the absence of population based and nationally representative studies on various clinical causes of anaemia raises the question whether the lack of intervention impact is due to the faulty methodology used for the determination of Hb level.

**Table 1.** Change in prevalence of anaemia among children (aged 6–59 months), pregnant women (aged 15–49 years), non-pregnant women (aged 15–49 years), and men (aged 15–49 years), during 2005–2006, 2015–2016, and 2019–2021

States/Union Territories	Children (Hb: <11 g/dl)			Pregnant women (Hb: <11 g/dl)			Non-pregnant women (Hb: <12 g/dl)			Men (Hb: <13 g/dl)		
	2005–2006	2015–2016	2019–2021*	2005–2006	2015–2016	2019–2021*	2005–2006	2015–2016	2019–2021*	2005–2006	2015–2016	2019–2021*
Andhra Pradesh (including Telangana)†	70·9	59·5	**	58·2	50·9	**	63·1	58·8	**	23·3	22·2	**
Arunachal Pradesh	58·1	54·4	56·6	51·8	37·8	27·9	50·6	43·5	40·8	27·7	18·8	21·4
Assam	69·0	35·9	68·4	72·0	44·8	54·2	69·1	46·1	66·4	39·4	25·6	36·0
Bihar	78·0	63·5	69·4	60·2	58·3	63·1	68·2	60·4	63·6	34·3	32·8	29·5
Chhattisgarh	72·1	41·7	67·2	63·1	41·5	51·8	57·1	47·3	61·2	27·0	22·1	27·0
Delhi	57·1	59·9	69·2	29·9	46·1	42·2	45·0	54·7	50·2	17·8	21·6	12·6
Goa	38·8	48·4	53·2	36·9	26·7	41·0	37·9	31·4	38·9	10·4	10·9	12·0
Gujarat	70·0	62·4	79·7	60·8	51·3	62·6	55·2	55·1	65·1	22·2	21·9	26·6
Haryana	72·5	71·8	70·4	69·7	55·0	56·5	55·2	63·1	60·6	19·2	21·2	18·9
Himachal Pradesh	54·0	53·7	55·4	38·1	50·4	42·2	43·2	53·6	53·4	18·5	20·3	18·6
Jammu & Kashmir (including Ladakh)‡	58·9	54·2	**	55·7	47·5	**	51·9	49·5	**	19·4	20·7	**
Jharkhand	70·5	70·1	67·5	68·5	62·6	56·8	69·4	65·3	65·7	36·5	30·2	29·6
Karnataka	70·6	61·3	65·5	60·4	45·4	45·7	50·8	44·8	47·8	19·0	18·2	19·6
Kerala	44·6	36·0	39·4	33·8	22·6	31·4	32·8	34·7	36·5	8·0	11·6	17·8
Madhya Pradesh	73·9	69·0	72·7	57·9	54·6	52·9	55·8	52·4	54·7	25·4	25·5	22·4
Maharashtra	63·3	54·0	68·9	57·8	49·3	45·7	48·0	47·9	54·5	16·8	17·6	21·9
Manipur	41·6	24·0	42·8	36·3	26·0	32·4	35·7	26·4	29·3	11·4	9·3	6·0
Meghalaya	64·7	48·1	45·1	58·1	53·3	45·0	45·4	56·4	54·4	36·5	32·2	25·5
Mizoram	44·1	19·6	46·4	48·3	27·0	34·0	37·6	24·7	34·8	19·4	12·5	15·6
Nagaland	§	26·6	42·7	§	32·7	22·2	§	27·7	29·3	§	11·7	10·0
Odisha	65·4	44·6	64·2	68·1	47·6	61·8	60·9	51·2	64·4	33·9	28·3	28·5
Punjab	66·2	56·7	71·1	41·6	42·0	51·7	37·9	54·0	58·8	13·6	25·9	22·6
Rajasthan	70·6	60·4	71·5	61·7	46·6	46·3	52·6	46·8	54·7	23·6	17·2	23·2
Sikkim	57·3	55·5	56·4	62·1	23·6	40·7	59·4	35·2	42·1	24·7	15·7	18·7
Tamil Nadu	63·6	50·8	57·4	54·7	44·4	48·3	53·1	55·4	53·6	16·6	20·6	15·2
Tripura	63·2	48·2	64·3	57·6	54·4	61·5	65·6	54·5	67·4	35·5	25·1	36·9
Uttar Pradesh	74·0	63·4	66·4	51·5	51·0	45·9	49·7	52·5	50·6	24·3	23·7	21·5
Uttarakhand	61·1	60·2	58·8	50·8	46·5	46·4	54·8	45·1	42·4	28·7	15·6	15·1
West Bengal	61·0	54·5	69·0	62·6	53·6	62·3	63·2	62·8	71·7	32·3	30·6	38·9
Andaman & Nicobar Islands	nc	50·3	40·0	nc	61·4	53·7	nc	65·8	57·6	nc	31·2	16·1
Chandigarh	nc	72·2	54·6	nc	76·8	dna	nc	75·9	60·1	nc	19·3	8·1
Dadra & Nagar Haveli	nc	84·4	***	nc	67·9	***	nc	80·1	***	nc	31·1	***
Daman & Diu	nc	74·5	***	nc	39·7	***	nc	59·3	***	nc	23·4	***
Lakshadweep	nc	52·6	43·1	nc	39·0	20·9	nc	46·3	26·0	nc	11·8	5·6
Puducherry	nc	44·9	64·0	nc	26·0	42·5	nc	53·4	55·5	nc	15·8	19·5
Andhra Pradesh†	nr	58·5	63·2	nr	52·9	53·7	nr	60·2	59·0	nr	26·8	16·2
Telangana†	nr	60·6	70·0	nr	48·2	53·2	nr	56·9	57·8	nr	15·5	15·3
Jammu & Kashmir‡	nr	53·5	72·7	nr	46·9	44·1	nr	49·0	67·3	nr	20·4	36·7
Ladakh‡	nr	91·4	92·5	nr	79·3	78·1	nr	78·4	93·7	nr	40·6	75·6
India‖	69·5	58·7	67·1	57·9	50·4	52·2	55·2	53·2	57·2	24·2	22·9	25·0

dna, data not available; g/dl, grams/decilitre; NFHS, National Family Health Survey; nc, not collected; nr, not required.

* The 2019–2021 NFHS data for analysis are not available yet. Thus, Hb estimates provided are not adjusted for altitude or/and smoking.

† During 2005–2006 NFHS, separate estimates for Andhra Pradesh and Telangana were not available, whereas during 2015–2016 NFHS, separate data were collected for Andhra Pradesh and Telangana.

‡ Ladakh was integral part of Jammu & Kashmir during 2005–2006 NFHS, whereas 2015–2016 NFHS collected district-level information which made helped generate separate estimate for Ladakh and Jammu & Kashmir. The Union Territory Ladakh was established in 2019.

§ Hb level was not measured during 2005–2006 NFHS.

‖ In 2015–2016, the national estimates for anaemia did not vary between twenty-eight states/union territories (included in 2005–2006) and 37 states/union territories (included in 2015–2016).

** Since 2019–2021 NFHS data set is not available, estimates of anaemia for Andhra Pradesh (including Telangana) and Jammu & Kashmir (including Ladakh) were not provided.

*** The 2019–2021 NFHS data for analysis are not available yet. Estimates on anaemia level were sourced from state fact sheets available at: http://rchiips.org/nfhs/. However, separate estimates for Dadra & Nagar Haveli and Daman & Diu were not provided in 2019–2021 NFHS fact sheet.

Measured Hb level obtained from the capillary blood sample are adjusted for altitude among children, whereas Hb level for women and men are adjusted for altitude and smoking. Details about adjustment are available elsewhere.

**Fig. 1.**
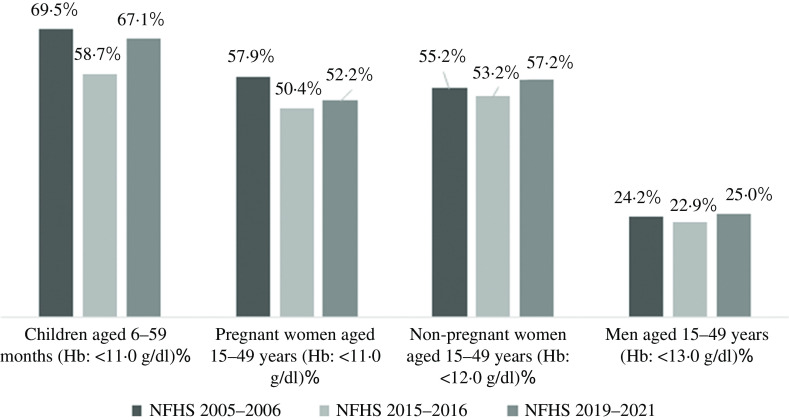
Prevalence (%) of anaemia among children, men and women in India, by age groups, National Family Health Survey (NFHS) 2005–2006, 2015–2016 and 2019–2021. Estimates of 2019–2021 NFHS are not adjusted for the altitude or/and smoking as the data for analysis are not available yet. g/dl, gram/deciliter.

To combat overall anaemia, the Indian government has introduced various national anaemia control programmes of Fe supplementation, which defined beneficiaries and pharmacological dose for Fe intervention (Table 2)^(39)^. In 1970, National Nutritional Anaemia Prophylaxis Programme was launched. Following an evaluation of the National Nutritional Anaemia Prophylaxis Programme in 1989 by the Indian Council of Medical Research, the apex public body for making India’s public health policy, an expert group meeting organised by the Ministry of Health and Family Welfare recommended that the dose of Fe for adults may be increased from 60 mg to 100 mg elemental Fe, thus in 1991 the National Nutritional Anaemia Control Programme (NNACP) was born. After over 20 years of silence, in 2012, the Weekly Iron and Folic Acid Supplementation programme was developed focusing on adolescents, especially adolescent girls. After much deliberation over the failure of anaemia control programmes, the National Iron Plus Initiative^(6)^ was launched in 2013, reiterating the need for interventions targeting children, adolescents, women in reproductive age group and pregnant and lactating women. The 2017 National Health Policy of India heavily emphasised the need for screening and treatment of anaemia^(40)^. However, five decades of programming and policy to prevent and treat anaemia have borne little success. To push existing initiatives, the Anaemia *Mukt Bharat* (Anaemia Free India) movement was launched in September 2018 by the current Prime Minister under the National Nutrition Mission. In addition to leveraging many of the aforementioned programmes, the Anaemia Free Movement calls for prophylactic iron-and-folic-acid (IFA) supplementation, administering de-worming medication, social and behaviour change communication campaigns, provisioning delayed cord clamping for new-borns, anaemia measurement at government health sub-centres (the lowest tier of public health care facility), mandatory provision of IFA fortified foods in public-funded programmes and addressing non-nutritional causes of anaemia with special focus on malaria, haemoglobinopathies and fluorosis^(40)^. Current anaemia reduction programmes heavily focus on pharmacological interventions of IFA tablet/syrup supplementation, even though the WHO estimates that anaemia among only 42 % (95 % CI: 38–46 %) of children aged 6–59 months, 49 % (95 % CI: 43–53 %) of non-pregnant women and 50 % (95 % CI: 47–53 %) of pregnant women aged 15–49 years is amenable to Fe supplementation^(5)^. In addition, the share of measured Fe deficiency in the aetiology of anaemia is lower in countries where the prevalence of anaemia is more than 40 %, such as India^(41)^.

**Table 2.** Evolution of national anaemia control programmes of iron supplementation

Programme	Inception year	Beneficiaries	Iron intervention/dose
National Nutritional Anaemia Prophylaxis Programme (NNAPP)	1970	• Children aged 1–5 years • Pregnant women • Lactating women • Intrauterine device (IUD) acceptors	• Children aged 1–5 years: 20 mg elemental iron and 100 mcg of folic acid (daily for 100 d) • Pregnant women, lactating women and IUD acceptors: 60 mg elemental iron and 500 mcg of folic acid
National Nutritional Anaemia Control Programme (NNACP)	1991	• Children aged 1–5 years • Pregnant women • Lactating women • Intrauterine device (IUD) acceptors	• Children aged 1–5 years: 20 mg elemental iron and 100 mcg of folic acid • Pregnant women, lactating women and IUD acceptors: 100 mg elemental iron and 500 mcg of folic acid.
Weekly Iron and Folic Acid Supplementation (WIFS) programme	2012	• School adolescent boys and girls (aged 10–19 years) of grade 6 through 12, enrolled in government/government aided/municipal schools • Out of school girls (aged 10–19 years)	• 100 mg elemental iron and 500 mcg of folic acid
National Iron Plus Initiative (NIPI)	2013	• Children aged 6–59 months • Children aged 5–9 years • Adolescents aged 10–19 years (in school boys and girls and out of school girls) • Women in reproductive age group (15–49 years) • Pregnant women • Lactating women	• Children aged 6–59 months: 1 ml of IFA syrup containing 20 mg of elemental iron and 100 mcg of folic acid • Children aged 5–9 years: Tablets of 45 mg elemental iron and 400 mcg of folic acid • Adolescents aged 10–19 years (in school boys and girls, and out of school girls), women in reproductive age group (15–49 years), pregnant women and lactating women: 100 mg elemental iron and 500 mcg of folic acid
Anaemia *Mukt Bharat* (Anaemia Free India), Prime Minister’s Overarching Scheme for Holistic Nourishment (POSHAN) *Abhiyaan* (National Nutrition Mission)	2018	• Children aged 6–59 months • Children aged 5–9 years • Adolescents aged 10–19 years (in school boys and girls and out of school girls) • Women in reproductive age group (15–49 years) • Pregnant women • Lactating women	• Children aged 6–59 months: 1 ml of IFA syrup containing 20 mg of elemental iron and 100 mcg of folic acid • Children aged 5–9 years: Tablets of 45 mg elemental iron and 400 mcg of folic acid • Adolescents aged 10–19 years (in school boys and girls, and out of school girls), women in reproductive age group (15–49 years), pregnant women and lactating women: 60 mg elemental iron and 500 mcg of folic acid

mg, milligram; mcg, microgram.

This review article discusses existing public intervention strategies – both food-based and clinical – to tackle anaemia burden and argues that the approach to combat overall anaemia in India is still premature and might not be adequate to achieve India’s anaemia reduction goal by 2022 under the Anaemia Free India initiative. The current strategy may even hurt the target set by the World Health Assembly of 50 % reduction of anaemia in women of reproductive age by 2025. Considering the limitations of current anaemia control strategies, three pragmatic approaches are proposed to advance India’s anaemia reduction targets.

## Food-based strategies

One of the pathways to curb anaemia is through appropriate dietary intake. Several public programmes in India already exist to improve nutrition either through food supplementation or by providing financial support. India has five main social security programmes^(42)^ – social security pensions; the Mid-day Meal (MDM) programme, the Integrated Child Development Scheme (ICDS) and the Public Distribution System (PDS), which are legal entitlements under the 2013 National Food Security Act (NFSA); and the National Rural Employment Guarantee Act, which entitles individuals in rural India to 100 d of work^(42)^.

Of these social security programmes, social security pensions and National Rural Employment Guarantee Act offer exclusively financial support that can improve access to food and diversify food baskets for households. However, the question remains on whether financial support through these programmes can help tackle micronutrient deficiencies such as anaemia. Over the years, problems of corruption, lack of timeliness in payments, and poor coverage of the benefits and beneficiaries, often due to administrative bottlenecks and minimal accountability have been reported^(43)^, which may have hindered progress in anaemia control programmes. A strong monitoring and evaluation framework of public intervention programmes is needed to minimise corruption and to increase accountability. Furthermore, improved financial status does not always translate to improved food consumption patterns as households may have other priorities over nutrition or they lack the awareness of achieving adequate nutrition beyond staple grains^(44)^.

The MDM, ICDS and PDS directly provide fresh cooked meals, nutrition supplementation and subsidised food grains, respectively, to beneficiaries. In Schedule II of the NFSA, nutritional standards (type of meal, required energies and protein intake) for children in the age groups of 6 months to 3 years, 3 to 6 years, pregnant women and lactating mothers (Table 3) are outlined^(45)^. The PDS provides subsidised monthly rations of rice or/and wheat to targeted households, as well as subsidised oil, sugar and salt^(46)^. Few states offer additional commodities such as pulses and legumes. The ICDS provides nutrition supplementation through fresh-cooked meals for children aged 3–6 years and through take home nutrition supplementation for children aged 6 months to 3 years, lactating mothers and pregnant women. The MDM, which though introduced as an intervention to boost school participation, has since become a key tool to tackle nutrition for school-going children through free lunches at public schools.

**Table 3.** The nutritional standards for children in the age group of 6 months to 3 years, age group of 3 to 6 years and pregnant women and lactating mothers required to be met by providing ‘take home rations’ or nutritious hot cooked meal in accordance with the integrated Child Development Services Scheme and nutritional standards for children in lower and upper primary classes under the Mid-Day Meal Scheme, The National Food Security Act, 2013

Serial number	Category	Type of meal	Calories (kcal)	Protein (g)
1	Children (6 months to 3 years)	Take home ration	500	12–15
2	Children (3 to 6 years)	Morning snack and hot cooked meal	500	12–15
3	Children (6 months to 6 years) who are malnourished	Take home ration	800	20–25
4	Lower primary classes	Hot cooked meal	450	12
5	Upper primary classes	Hot cooked meal	700	20
6	Pregnant women and lactating mothers	Take home ration	600	18–20

g, gram; kcal, kilocalorie.

Ministry of Law and Justice (2013) The National Food Security Act, 2013. The Gazette of India, Government of India, New Delhi.

The MDM has been among the more successful of India’s social programmes by ensuring one nutritious meal for school going children each day^(42)^. A recent study has estimated that between 2006 and 2016, MDM was associated with 13–23 % of the height-for-age improvement^(47)^, but whether MDM has been able to reduce the burden of anaemia among children remains to be explored. The PDS and ICDS have made great strides over the years in improving quality, transparency, accountability and coverage of food and nutrition benefits. However, administrative challenges still exist with a need for greater accountability^(48)^ improved quality and diversity of food being provided^(49)^ and eliminating exclusion of vulnerable households who most sorely need these benefits^(50)^. Furthermore, coverage of these programmes leaves much to be desired – only 52·3 % of all households had eligibility to purchase cereals from the PDS in 2011–2012^(51)^. Therefore, these are not fool-proof delivery systems for nutrition goods and services.

Government food and nutrition interventions have traditionally focused on protein energy malnutrition, leaving behind micronutrient deficiencies such as IDA. To meet the goals of the Anaemia Free India movement, one strategy being explored by the government is to distribute fortified food grains, and the Department of Food and Public Distribution, Government of India has launched a centrally sponsored pilot scheme on rice fortification in the PDS, covering fifteen districts^(52)^. The target concentrations for fortification of different nutrients in several food staples have been notified in the Indian Gazette in 2018^(53)^, and fortification has been made mandatory in 2020^(54)^. As per the centrally sponsored pilot scheme on ‘Fortification of Rice and its Distribution under Public Distribution System’ approved on February 14, 2019, Fe, vitamin B_12_ and folic acid are the mandatory nutrients to be used for the fortification of rice^(55)^ and wheat^(56)^. Addressing IDA using fortified rice has a history of mixed results. One study conducted with Cambodian schoolchildren using several formulations found no significant Hb change in 6 months^(57)^. A recent Cochrane review with meta-analysis^(58)^ found fortification of rice with Fe (alone or in combination with other micronutrients) may make little or no difference in the risk of having anaemia and the authors were uncertain about an increase in mean Hb concentrations in the general population above 2 years of age.

There is potential for Fe fortification of rice in India since it is widely consumed. However, the bio-availability issues would need to be solved, and continued scientific research^(59)^ on improved Fe bioavailability in rice and support for additional research could be a globally beneficial exercise. Progress has been made, but some of the developments are of special patented Fe forms possibly less suitable for public health programmes^(60)^. The public supply programmes for wheat are a different matter. Although there are complexities to each of the different programmes, it is well known how to fortify wheat flour to dramatically reduce Fe deficiency and IDA. Implementing this fortification has its own challenges since a significant quantity of the wheat provided under the PDS is given as grain which is then milled in small mills to wheat flour. Setting up fortification programmes at thousands of mills is a major logistical, financial and quality control undertaking.

To the extent that the PDS can deliver wheat flour from large-scale flour mills rather than whole grains, this wheat flour could be fortified immediately. However, to be effective, the fortification would have to follow the guideline developed after extensive review by global experts for the WHO^(61)^. These experts found only one form of Fe (iron sodium ethylenediaminotetraacetate or FeNaEDTA) was sufficiently bioavailable in the presence of the high phytic acid levels present in whole wheat flour, to be useful in reducing Fe deficiency or IDA. In a randomised, double-blind, controlled school feeding trial^(62)^, wheat flour was fortified with FeNaEDTA and the study showed reduction in Fe deficiency in 7 months by 66 % and IDA by 50 %. This form of Fe is permitted for fortification in India but not widely used so far, presumably due to lack of local production and relatively high cost. However, significant funds have been wasted fortifying wheat flour with forms of Fe that are insufficiently bioavailable to reduce Fe deficiency or IDA. The MDM programmes could probably implement such effective fortification quickly if budgets were authorised, and public advocacy might help this process along. In recent developments, the Government of India is considering use of millets in the MDM programme. A systematic review and meta-analysis^(63)^ suggested that finger millet, pearl millet, teff and sorghum or a mixture of millets could help in reducing IDA. A randomised controlled trial^(64)^ conducted among children in India found that Fe-biofortified pearl millet could significantly reduce childhood anaemia.

The ICDS programme has already been active in the distribution of IFA tablets, as well as supplementary nutrition packets for women and children that are micronutrient fortified and provide protein. As nearly half of India’s anaemia cases are linked to Fe deficiency^(5)^, food-based programmes such as the PDS, ICDS and MDM might not be helpful in addressing overall anaemia. In addition, although the effect of fortified food through PDS on anaemia reduction is yet to be realised, it is unlikely that targeted PDS would be able to make a dent at the population level.

In the context of a food-based strategy for anaemia reduction, the bioavailability of Fe seems to be poorly understood. People in India largely derive their dietary Fe from non-haem, inorganic sources, including grains, plants, cereals, lentils and vegetables, as compared with sources of haem Fe such as meat and fish, which have a higher rate of absorption^(65)^. Red meat is also an effective source for bioavailable Fe, and recommendations for increased consumption could be a strategy among the non-vegetarian segment of the population, but this may be infeasible from a political point of view considering religious sensitivities and the relatively high cost. It is not clear how the government plans, if at all, to blend these interventions as per the dietary habits of the population. However, it is encouraging to note that per capita consumption of dairy, meat, vegetables and fruit is projected to increase, with the magnitude of the projected increase in meat and dairy consumption expected to be directly related to income growth^(66)^. Also, in the intervention guideline spearheaded by the Department of Health and Family Welfare, Government of India, there is no strict guideline about when to stop consuming Fe supplements. Overconsumption of IFA may alter gut microbiota composition that may affect overall health, such as overgrowth of pathogenic bacteria which could lead to adverse health outcomes in long terms, such as colon cancer^(67)^. These studies are not conclusive and linking food consumption with cancer has been challenged by researchers^(68)^. In addition, the Government of India does not provide any guidelines for IFA consumption for more effective Fe absorption in the gut. A person with sub-clinical inflammation may have poor absorption of Fe that should be treated before administering Fe^(69)^. Recently, a group of researchers warned the Government of India that layering of multiple micronutrients in fortification programs and incorrect biomarker cut-offs to evaluate nutrient deficiencies in the population is unnecessary and could be harmful^(70)^.

## Clinical strategies

In addition to food-based strategies for improving Fe intake and reducing IDA, numerous clinical strategies have also been outlined to address anaemia. However, various research studies and reports indicate an over-reliance of government programmes on pharmacological intervention of prophylactic IFA supplementation to mitigate IDA, especially among women and children, which may not result in anaemia reduction^(31)^. A recent randomised placebo-controlled trial in Bangladesh^(71)^ concluded that 3 months of daily supplementation with Fe syrup or multiple micronutrient powders had no effect on child development or other functional outcomes compared with infants who received a placebo. Weekly Iron and Folic Acid Supplementation programme currently focuses on adolescents aged 10–19 years guided by the National Iron Plus Initiative, which provides guidelines for the overall IFA intervention strategy. The Ministry of Health and Family Welfare has tried to increase the bioavailability of IFA by planning to introduce sugar-coated IFA tablets instead of enteric coated tablets^(72)^. IFA uptake among women and children has been low for several reasons^(13)^. According to the 2019–2021 NFHS, 44·1 % women consumed ≥100 IFA tablets or equivalent syrup during their last pregnancy, which is a substantial improvement from 2005–2006 NFHS (15·2 %) and 2015–2016 NFHS (30·3 %), whereas during 2015–2016 only 25·3 % children received any IFA tablet or syrup in the 7 d preceding the survey date (Table 4), varying widely across the 640 districts of India (data not shown separately). Although these are considered an improvement over the 2005–2006 NFHS data (Table 4), it shows that the outreach of the initiative is still poor and has had almost no effect on reduction of overall anaemia. This raises the question as to what Anaemia *Mukt Bharat* would do differently if IFA distribution remains the same as earlier initiatives.

**Table 4.** Change (2005–2006, 2015–2016 or/and 2019–2021) in receipt of weekly iron tablet or syrup supplementation (WIS) by children (aged 0–59 months) preceding 7 d of survey date and women (aged 15–49 years) who consumed ≥100 d of iron & folic acid (≥100 IFA) tablets or equivalent syrup for their recent pregnancy preceding 5 years of survey date

States/Union Territories	WIS		≥100 IFA		
	2005–2006	2015–2016	2005–2006	2015–2016	2019–2021
Andhra Pradesh (including Telangana)†	7·0	32·1	26·3	54·7	*
Arunachal Pradesh	3·9	20·3	7·3	8·3	23·8
Assam	0·8	19·8	10·3	32·0	47·5
Bihar	2·7	21·0	6·3	9·7	18·0
Chhattisgarh	3·4	33·8	10·3	30·3	45·0
Delhi	8·8	26·5	27·5	53·9	69·1
Goa	17·1	54·5	59·6	67·4	87·5
Gujarat	10·3	31·0	25·3	36·8	60·0
Haryana	4·0	39·4	17·7	32·5	51·2
Himachal Pradesh	3·9	18·9	25·2	49·4	67·2
Jammu & Kashmir (including Ladakh)‡	4·9	18·6	16·3	30·2	*
Jharkhand	3·3	16·9	9·5	15·3	28·2
Karnataka	12·2	49·1	28·2	45·3	44·7
Kerala	6·4	17·5	70·1	67·1	80·0
Madhya Pradesh	3·4	25·1	7·1	23·6	51·4
Maharashtra	7·4	40·2	18·6	40·6	48·2
Manipur	2·2	4·3	6·8	39·2	52·3
Meghalaya	5·3	28·8	5·9	36·2	43·1
Mizoram	20·8	23·4	17·8	53·6	61·9
Nagaland	2·9	7·7	1·2	4·4	10·2
Odisha	5·5	26·7	22·5	36·5	60·8
Punjab	4·9	31·3	13·2	42·6	55·4
Rajasthan	0·9	13·9	8·7	17·3	33·9
Sikkim	9·3	48·4	26·3	52·8	54·7
Tamil Nadu	9·9	32·9	28·2	64·0	82·5
Tripura	2·9	7·3	11·5	13·4	26·7
Uttar Pradesh	1·5	12·8	6·0	12·9	22·3
Uttarakhand	3·8	13·8	16·4	24·9	46·6
West Bengal	4·3	26·3	14·3	28·0	62·5
Andaman & Nicobar Islands	nc	23·3	nc	58·4	80·9
Chandigarh	nc	11·8	nc	44·9	73·9
Dadra & Nagar Haveli	nc	14·5	nc	43·9	**
Daman & Diu	nc	25·0	nc	38·3	**
Lakshadweep	nc	10·4	nc	81·7	80·1
Puducherry	nc	43·2	nc	66·3	84·1
Andhra Pradesh†	nr	28·2	nr	56·2	70·3
Telangana†	nr	36·9	nr	52·8	57·9
Jammu & Kashmir‡	nr	18·8	nr	30·2	29·8
Ladakh‡	nr	9·6	nr	29·5	14·3
India§	4·6	25·3	15·2	30·3	44·1

NFHS, National Family Health Survey; na, not available; nc, not collected; nr, not required.

* The 2019–2021 NFHS data for analysis are not available yet. Estimates on ≥100 IFA consumption were sourced from state fact sheets available at: http://rchiips.org/nfhs/. Thus, estimates for Andhra Pradesh (including Telangana) and Jammu & Kashmir (including Ladakh) were not possible to calculate.

† During 2005–2006 NFHS, separate estimates for Andhra Pradesh and Telangana were not available, whereas during 2015–2016 NFHS, separate data were collected for Andhra Pradesh and Telangana.

‡ Ladakh was integral part of Jammu & Kashmir during 2005–2006 NFHS, whereas 2015–2016 NFHS collected district-level information which made helped generate separate estimate for Ladakh and Jammu & Kashmir. The Union Territory Ladakh was established in 2019.

§ In 2015–2016, the national estimates for anaemia did not vary between twenty-eight states/union territories (included in 2005–2006) and thirty-seven states/union territories (included in 2015–2016).

** The 2019–2021 NFHS data for analysis are not available yet. Estimates on ≥100 IFA consumption were sourced from state fact sheets available at: http://rchiips.org/nfhs/. However, separate estimates for Dadra & Nagar Haveli and Daman & Diu were not provided in 2019–2021 NFHS fact sheet.

The 2019–2021 NFHS data for analysis are not available yet. Thus, estimate of WIS during 2019–2021 was not estimated.

The burden of helminths is an important contributor to anaemia in India. A national study found nearly 50 % of India’s population living across six states had helminths^(73)^, and a recent meta-analysis study reported that incidence of anaemia often coincided with infection, with Hb levels ranging from 8·2 to 11·02 g/dl^(74)^. While the Weekly Iron and Folic Acid Supplementation programme in India includes albendazole to treat helminths among adolescents aged 10–19 years, the NIPI programme^(6)^ recommends albendazole to children (aged 1–10 years) and women in reproductive age group (15–45 years), with different dose and regime. A programme to greatly reduce helminth-caused anaemia must be an important component of anaemia reduction in India.

Interventions to tackle the exceedingly high burden of fluorosis (referring to the mineral calcium fluoride) remain negligible in India, where it is found in high concentrations both in untreated groundwater and popular foods^(75)^. In addition, the Ministry of Health and Family Welfare of India has estimated that India has the largest number of children with Thalassemia major in the world – about 0·1 to 0·15 million with almost 42 million carriers of the *β* (beta) thalassemia trait. Sickle cell disease has been found in the states of Gujarat, Maharashtra and Kerala at rates of 1–3 %. Globally, nearly 450 000 infants are born with haemoglobinopathies. While studies have shown that the most common haemoglobinopathy in India is *α*-thalassemia, the prevalence of *β*-thalassemia in India is estimated to be nearly 3·3%^(76)^. The Ministry of Health and Family Welfare guideline on anaemia prevention^(77)^ is a step forward, but universal screening has not been implemented in India yet. This would identify homozygotes for medical treatment, as well as find heterozygote carriers who may be at risk, and for whom anaemia prevention could be addressed directly. Several strategies^(78)^ are available and the regional experience in Sri Lanka could be instructive^(79)^. An advocacy effort might work to gain support for furthering research and development into rapid screening which could test for haemoglobinopathies and Fe deficiency in one test, as demonstrated in a recent study conducted in Sri Lanka^(80)^.

According to the WHO, ‘delayed umbilical cord clamping (not earlier than 1 min after birth) is recommended for improved maternal and infant health and nutrition outcomes’^(81)^. It would allow blood flow between the placenta and neonate to continue, which may improve Fe status in infants for up to 6 months after birth. Although this measure appears attractive, the benefits should be weighed in terms of possible harmful effects^(82–84)^. In India, there is no evidence that any systematic procedure for cord clamping is practiced by personnel assisting in delivery.

The 2017 National Health Policy indicated screening of anaemia at the primary healthcare level. Studies show there is no systematic practice for screening anaemia, and identification of anaemic individuals is often by accident. Non-nutritional causes of anaemia such as helminths, malaria, haemoglobinopathies and fluorosis are barely addressed in anaemia reduction programmes. Also, serum ferritin should work as exemplified by low Hb with high ferritin, but the problem is that this process is not suitable for screening. The National Institute of Nutrition, an apex public body of the Indian Council of Medical Research responsible for devising national nutrition policies, is currently assessing the effectiveness of population-level screening followed by targeted IFA supplementation (according to the grade of anaemia) with an aim to increase the mean population-level Hb and thereby reduce the prevalence of anaemia. Studies like this will be able to shed light on India’s need for a screen and treat approach for anaemia control.

### Conclusions

With these two strategic lapses by the Indian government, programmes are still emphasised on IDA, and despite improvements in social security programmes, problems of access, exclusion and leakage remain concerns. In light of the above discussion, this review proposes a threefold strategy for consideration.

First, it is essential for the Indian government to undertake a nation-wide survey to estimate the true burden of anaemia, and district-level estimates are highly recommended. The Government of India should undertake a survey that would collect venous blood samples to measure Hb concentration. In addition, the nationwide anaemia survey must attempt to estimate the causes of anaemia in India. India does not have an estimate of clinical determinants of anaemia, which is essential for devising any intervention for anaemia control. Conducting a nationwide survey to measure Hb level and its determinants from venous blood sample is expensive and a logistic-intensive effort. The 2016–2018 CNNS could be a reference and guideline to conduct a nationwide survey. CNNS measured anaemia, IDA and micronutrient deficiency (vitamin A, vitamin D, Zn deficiency, vitamin B_12_ and folate deficiency and urinary iodine status) using whole blood, which required sending blood for a standardised laboratory test. Similarly, in a phase-wise manner, conducting a nationwide test for Hb levels and related biomarker tests should not be an impossible task for the Indian government. As outlined in the CNNS survey, measurement of anaemia was adjusted for various biochemical parameters such as inflammation. Anaemia level is recommended to be adjusted for the inflammation level to understand the real burden of anaemia in an individual^(85–87)^.

Second, although inconclusive, a food-based strategy could be a better solution for Fe-deficiency anaemia reduction^(88)^, which calls for improving dietary diversity through the availability and affordability of high quality^(70)^. There is mixed evidence on the effect of bio-fortified food and its effect on anaemia control. A study concluded that Fe-biofortified pearl millet may help reduce the burden of anaemia among adolescents^(89)^ in India, however, a recent systematic review of Fe biofortification written by many of the same authors of the pearl millet intervention and^(90)^ found ‘no significant effects on categorical outcomes such as Fe deficiency or anaemia’. The health status of the individual is key to absorb and mobilise Fe, which means that individuals with infection or inflammation would see less improvement even with the provision of additional Fe. This discussion calls for further research on this issue. Development of a well-organised food-based strategy, especially improving habitual diets, concentrating more on meal consumption patterns and food constituents may be one of the keys in modifying Fe bioavailability, thus reducing overall anaemia burden. Also, an excess cost of Fe supplementation through mandatory food fortification would pose a huge burden to India’s economy^(70)^ As per recent estimates, Fe fortified of rice alone costs ∼$350 million/year which is expected to increase. This cost excludes the budget of ∼$130 million reserved for anaemia *Mukt Bharat* programme during 2019–2020^(70)^.

In addition, it is unknown why the government is solely dependent on IFA tablet/syrup intervention when a true estimate of anaemia in India is unknown and clinical causes of anaemia beyond Fe deficiency are overlooked, which calls for further research on benefit and side effects of IFA supplementation to decide required dose of Fe. Alternative forms of Fe for supplementation have also been poorly studied. This is likely due to the relatively low profit margins for Fe compounds other than for pharmaceutical use. A study in India^(91)^ showed FeNaEDTA (the only type of Fe recommended by WHO/FAO for fortification of high extraction flour) was at least three times as effective as ferrous fumarate in supplementation studies (that is to say, 1/3 the dose of FeNaEDTA was as effective as a full daily dose of 200 mg ferrous fumarate). There are indications that even lower FeNaEDTA supplementations could be equally effective. Support for research into these alternatives could be globally beneficial.

Third, there is discussion on the need for a revision of cut-offs of Hb concentration^(92)^, and the WHO has convened several meetings on this topic^(92)^. A recent study in India using 2016–2018 CNNS data demonstrated the need for a revision of the Hb threshold for the Indian population aged 1–19 years^(92)^. Before leaping into more interventions to tackle the anaemia burden in India, policymakers and practitioners would benefit to await the recommendations of these proceedings.

In addition, there are some serious issues to be considered while devising intervention strategies. There appears to be a U-curve for Fe supplementation^(93)^, which may be partially ameliorated by weekly *v*. daily intakes^(94)^. India has several different supplementation programmes, some of which rely on weekly rather than daily intake^(39)^. It is worth mentioning that the U-shaped relation could vary by trimester for pregnant women, and there is less evidence for the associations between maternal Fe status and adverse birth outcomes, which calls for further research on Hb cut-offs used for vulnerable groups. In addition, the type of Fe supplement used can lead to sharp increases in non-transferrin-bound Fe^(95,96)^. There is evidence that non-transferrin-bound Fe may contribute to malaria severity^(97)^ though not yet conclusively demonstrated, and non-transferrin-bound Fe has been linked to biomarkers of oxidative stress, inflammation and endothelial dysfunction in type 2 diabetes^(91)^. There are some operational issues in the IFA programmes attempting to reach over 130 million adolescents and an additional 16 million pregnant women. This effort is an enormous logistical, educational and managerial challenge that ultimately relies on compliance from the subjects.

To conclude, while the WHO is reviewing the anaemia threshold, without introducing any new anaemia control programme, the Indian government should encourage the food fortification with FeNaEDTA to help correct Fe deficiency and IDA among the Indian population. In parallel, the government of India should prioritise conducting a nationwide survey on estimation of true burden of anaemia from venous blood samples. Vulnerable groups such as pregnant and lactating women and children should be given special attention for measuring their Hb level at the secondary and tertiary care level, and if found anaemic according to existing guideline of WHO, further examination must be considered to determine the exact causes of anaemia. Once WHO releases the revised criteria to define anaemia and estimates on causes of anaemia in India are available, a checklist for required dose of Fe for an individual could be formulated depending on the need and disease status of an individual. The formulation of a checklist algorithm to guide individualised anaemia treatment strategy would likely require further research. Similarly, the effectiveness of food fortification programmes (with or without FeNaEDTA) demands further research on its costs and benefits to treat anaemia among Indians.

The anaemia *Mukht Bharat* initiative would require a comprehensive population-based strategy that is absent in its current form. The current national strategy lacks an adequate scientific approach for measuring both the actual burden and determinants of anaemia. Over 50 years of national policy on anaemia can be best described as shooting shadows, with overemphasis on food fortification and IFA supplementation and little heed to the complexity surrounding the incidence of anaemia. Recent research has shown promising interventions and opportunities to apply a holistic approach to treat anaemia as well as questioned the standards by which anaemia is currently measured. Going forward, national anaemia reduction policy must be rooted in proven scientific measures that are sensitive to the Indian setting and address the multiple entry points for anaemia in an individual’s life, as outlined in this review.
